# Supplementary material for: Effects of Lactobacillus plantarum Q180 on Postprandial Lipid Levels and Intestinal Environment: A Double-Blind, Randomized, Placebo-Controlled, Parallel Trial
Source: Nutrients. 2020 Jan 19;12(1):255. doi: 10.3390/nu12010255 (PMC7019774; doi:10.3390/nu12010255)
Supplement: Supplementary file 1 [file nutrients-12-00255-s001.zip › Supplement table 3.docx]

| **Supplementary Table S3.** The overall correlation between baseline levels of fecal metabolites and changes in blood lipid markers for 12 weeks.^1^ | | | | | |
| --- | --- | --- | --- | --- | --- |
| Variables | | Placebo (n=31) | | LPQ 180 (n=31) | |
|  |  | r^2^ | *p* | r^2^ | *p* |
| Biogenic amines | Δ TG | -0.231 | 0.211 | -0.047 | 0.804 |
|  | Δ TC | 0.110 | 0.557 | -0.167 | 0.369 |
|  | Δ LDL-C | 0.256 | 0.164 | 0.051 | 0.787 |
|  | Δ HDL-C | -0.094 | 0.616 | -0.257 | 0.164 |
|  | Δ VLDL-C | -0.231 | 0.211 | -0.047 | 0.804 |
|  | Δ Chylomicron TG | -0.047 | 0.804 | -0.039 | 0.836 |
|  | Δ ApoB | 0.134 | 0.473 | -0.039 | 0.835 |
|  | Δ ApoB-48 | -0.177 | 0.366 | -0.104 | 0.578 |
|  | Δ ApoB-100 | 0.168 | 0.392 | -0.119 | 0.524 |
|  | Δ TG AUC | -0.243 | 0.204 | -0.348 | 0.104 |
|  | Δ TG C_max_ | -0.197 | 0.307 | -0.323 | 0.133 |
|  | Δ Chylomicron TG AUC | -0.205 | 0.287 | -0.312 | 0.129 |
|  | Δ Chylomicron TG C_max_ | -0.326 | 0.104 | -0.373 | 0.080 |
|  | Δ ApoB-48 AUC | -0.184 | 0.349 | -0.155 | 0.470 |
|  | Δ ApoB-48 C_max_ | -0.156 | 0.428 | -0.437 | 0.033 |
|  | Δ ApoB-100 AUC | 0.072 | 0.716 | 0.060 | 0.782 |
|  | Δ ApoB-100 C_max_ | 0.088 | 0.668 | 0.071 | 0.749 |
| Short chain fatty acids | Δ TG | -0.019 | 0.918 | -0.180 | 0.332 |
|  | Δ TC | -0.310 | 0.090 | -0.048 | 0.796 |
|  | Δ LDL-C | -0.531 | 0.002 | 0.021 | 0.912 |
|  | Δ HDL-C | -0.332 | 0.069 | 0.017 | 0.930 |
|  | Δ VLDL-C | -0.019 | 0.918 | -0.180 | 0.332 |
|  | Δ Chylomicron TG | 0.122 | 0.538 | 0.137 | 0.463 |
|  | Δ ApoB | -0.328 | 0.072 | -0.024 | 0.898 |
|  | Δ ApoB-48 | 0.036 | 0.857 | -0.104 | 0.579 |
|  | Δ ApoB-100 | -0.149 | 0.451 | 0.019 | 0.921 |
|  | Δ TG AUC | 0.108 | 0.577 | 0.097 | 0.659 |
|  | Δ TG C_max_ | 0.059 | 0.760 | 0.309 | 0.151 |
|  | Δ Chylomicron TG AUC | 0.230 | 0.230 | 0.360 | 0.078 |
|  | Δ Chylomicron TG C_max_ | 0.273 | 0.178 | 0.307 | 0.155 |
|  | Δ ApoB-48 AUC | 0.118 | 0.549 | -0.087 | 0.685 |
|  | Δ ApoB-48 C_max_ | 0.131 | 0.506 | 0.117 | 0.586 |
|  | Δ ApoB-100 AUC | -0.201 | 0.304 | -0.003 | 0.990 |
|  | Δ ApoB-100 C_max_ | 0.001 | 0.996 | 0.091 | 0.680 |
| Indole and phenols | Δ TG | 0.428 | 0.016 | -0.163 | 0.380 |
|  | Δ TC | 0.104 | 0.577 | -0.165 | 0.375 |
|  | Δ LDL-C | 0.085 | 0.650 | -0.067 | 0.719 |
|  | Δ HDL-C | 0.219 | 0.237 | 0.407 | 0.023 |
|  | Δ VLDL-C | 0.428 | 0.016 | -0.163 | 0.380 |
|  | Δ Chylomicron TG | 0.383 | 0.044 | -0.240 | 0.193 |
|  | Δ ApoB | 0.056 | 0.765 | 0.162 | 0.385 |
|  | Δ ApoB-48 | 0.275 | 0.156 | 0.086 | 0.646 |
|  | Δ ApoB-100 | 0.009 | 0.964 | -0.270 | 0.142 |
|  | Δ TG AUC | 0.377 | 0.044 | -0.047 | 0.832 |
|  | Δ TG Cmax | 0.480 | 0.009 | 0.120 | 0.585 |
|  | Δ Chylomicron TG AUC | 0.297 | 0.118 | -0.125 | 0.553 |
|  | Δ Chylomicron TG C_max_ | 0.399 | 0.044 | 0.038 | 0.862 |
|  | Δ ApoB-48 AUC | 0.497 | 0.007 | 0.130 | 0.546 |
|  | Δ ApoB-48 C_max_ | 0.489 | 0.008 | 0.119 | 0.580 |
|  | Δ ApoB-100 AUC | 0.085 | 0.667 | -0.149 | 0.488 |
|  | Δ ApoB-100 C_max_ | 0.064 | 0.756 | -0.134 | 0.541 |
| Neutral sterol | Δ TG | -0.057 | 0.763 | -0.281 | 0.126 |
|  | Δ TC | 0.082 | 0.662 | -0.148 | 0.426 |
|  | Δ LDL-C | 0.111 | 0.551 | -0.087 | 0.643 |
|  | Δ HDL-C | 0.099 | 0.596 | 0.252 | 0.171 |
|  | Δ VLDL-C | -0.057 | 0.763 | -0.281 | 0.126 |
|  | Δ Chylomicron TG | -0.095 | 0.632 | 0.048 | 0.798 |
|  | Δ ApoB | 0.114 | 0.541 | -0.132 | 0.478 |
|  | Δ ApoB-48 | -0.164 | 0.404 | -0.254 | 0.168 |
|  | Δ ApoB-100 | -0.111 | 0.575 | -0.308 | 0.092 |
|  | Δ TG AUC | -0.080 | 0.678 | -0.127 | 0.564 |
|  | Δ TG C_max_ | 0.087 | 0.654 | 0.052 | 0.814 |
|  | Δ Chylomicron TG AUC | -0.106 | 0.584 | 0.076 | 0.720 |
|  | Δ Chylomicron TG C_max_ | 0.193 | 0.344 | -0.029 | 0.897 |
|  | Δ ApoB-48 AUC | -0.068 | 0.731 | -0.249 | 0.241 |
|  | Δ ApoB-48 C_max_ | -0.025 | 0.901 | -0.096 | 0.657 |
|  | Δ ApoB-100 AUC | -0.065 | 0.742 | 0.074 | 0.730 |
|  | Δ ApoB-100 C_max_ | -0.083 | 0.686 | 0.069 | 0.756 |
| Primary bile acids | Δ TG | -0.242 | 0.190 | 0.200 | 0.280 |
|  | Δ TC | -0.005 | 0.977 | -0.057 | 0.762 |
|  | Δ LDL-C | 0.187 | 0.314 | -0.149 | 0.425 |
|  | Δ HDL-C | 0.040 | 0.829 | -0.097 | 0.602 |
|  | Δ VLDL-C | -0.242 | 0.190 | 0.200 | 0.280 |
|  | Δ Chylomicron TG | -0.276 | 0.155 | 0.012 | 0.947 |
|  | Δ ApoB | 0.126 | 0.499 | 0.007 | 0.970 |
|  | Δ ApoB-48 | -0.216 | 0.271 | 0.050 | 0.788 |
|  | Δ ApoB-100 | 0.008 | 0.969 | 0.054 | 0.771 |
|  | Δ TG AUC | -0.276 | 0.147 | 0.097 | 0.659 |
|  | Δ TG C_max_ | -0.278 | 0.144 | -0.232 | 0.288 |
|  | Δ Chylomicron TG AUC | -0.182 | 0.344 | -0.035 | 0.869 |
|  | Δ Chylomicron TG C_max_ | -0.211 | 0.302 | -0.140 | 0.524 |
|  | Δ ApoB-48 AUC | -0.113 | 0.569 | 0.025 | 0.906 |
|  | Δ ApoB-48 C_max_ | -0.095 | 0.632 | -0.072 | 0.738 |
|  | Δ ApoB-100 AUC | -0.025 | 0.900 | -0.079 | 0.713 |
|  | Δ ApoB-100 C_max_ | -0.350 | 0.079 | -0.170 | 0.438 |
| Secondary bile aicds | Δ TG | -0.242 | 0.190 | 0.200 | 0.280 |
|  | Δ TC | -0.005 | 0.977 | -0.057 | 0.762 |
|  | Δ LDL-C | 0.187 | 0.314 | -0.149 | 0.425 |
|  | Δ HDL-C | 0.040 | 0.829 | -0.097 | 0.602 |
|  | Δ VLDL-C | -0.242 | 0.190 | 0.200 | 0.280 |
|  | Δ Chylomicron TG | -0.276 | 0.155 | 0.012 | 0.947 |
|  | Δ ApoB | 0.126 | 0.499 | 0.007 | 0.970 |
|  | Δ ApoB-48 | -0.216 | 0.271 | 0.050 | 0.788 |
|  | Δ ApoB-100 | 0.008 | 0.969 | 0.054 | 0.771 |
|  | Δ TG AUC | -0.276 | 0.147 | 0.097 | 0.659 |
|  | Δ TG C_max_ | -0.278 | 0.144 | -0.232 | 0.288 |
|  | Δ Chylomicron TG AUC | -0.182 | 0.344 | -0.035 | 0.869 |
|  | Δ Chylomicron TG C_max_ | -0.211 | 0.302 | -0.140 | 0.524 |
|  | Δ ApoB-48 AUC | -0.113 | 0.569 | 0.025 | 0.906 |
|  | Δ ApoB-48 C_max_ | -0.095 | 0.632 | -0.072 | 0.738 |
|  | Δ ApoB-100 AUC | -0.025 | 0.900 | -0.079 | 0.713 |
|  | Δ ApoB-100 C_max_ | -0.350 | 0.079 | -0.170 | 0.438 |
| ^1^ LPQ 180, *Lactobacillus plantarum* Q180 ^2^ r: Pearson's correlation coefficients | | | | | |
