# Supplementary material for: Effects of Lactobacillus plantarum Q180 on Postprandial Lipid Levels and Intestinal Environment: A Double-Blind, Randomized, Placebo-Controlled, Parallel Trial
Source: Nutrients. 2020 Jan 19;12(1):255. doi: 10.3390/nu12010255 (PMC7019774; doi:10.3390/nu12010255)
Supplement: Supplementary file 1 [file nutrients-12-00255-s001.zip › Supplement table 1.docx]

| **Supplementary Table S1**. Comparison of intestinal microbial metabolites in feces for 12 weeks ^1^ | | | |
| --- | --- | --- | --- |
| Variables | Placebo  (n=35) | LPQ 180  (n=35) | *p*-value^2^ |
| **Biogenic amines (mg/g)** | | | |
| Agmatine | -0.35 ± 0.53 | -0.84 ± 0.55 | 0.641 |
| Methylamine | 0.18 ± 0.06 | -0.02 ± 0.06 | 0.102 |
| Ethylamine | 0.13 ± 0.21 | -0.41 ± 0.21 | 0.182 |
| Pyrrolidine | 0.46 ± 0.42 | -0.15 ± 0.44 | 0.461 |
| Dimethylamine | 0.09 ± 0.03 | -0.02 ± 0.03 | 0.105 |
| Tryptamine | 0.09 ± 0.04 | 0.01 ± 0.04 | 0.234 |
| Butylamine | 0.20 ± 0.29 | -0.06 ± 0.30 | 0.645 |
| Phenylethylamine | 0.37 ± 0.21 | 0.15 ± 0.22 | 0.608 |
| Putrescine | -0.13 ± 0.07 | -0.20 ± 0.08 | 0.662 |
| Cadaverine | 3.12 ± 1.92 | 1.96 ± 1.99 | 0.760 |
| Histamine | -0.16 ± 0.16 | -0.17 ± 0.17 | 0.959 |
| Tyramine | 0.20 ± 0.14 | -0.22 ± 0.15 | 0.145 |
| Spermidine | -0.49 ± 0.69 | -0.62 ± 0.71 | 0.928 |
| Spermine | -0.08 ± 0.09 | -0.04 ± 0.09 | 0.835 |
| Total Biogenic amines | 3.62 ± 2.91 | -0.62 ± 3.01 | 0.459 |
| **Short chain fatty acid (µg/g)** | | | |
| Acetic acid | 39.65 ± 22.23 | 0.52 ± 23.48 | 0.375 |
| Propionic acid | 9.85 ± 5.88 | -0.55 ± 6.21 | 0.373 |
| Butyric acid | -3.89 ± 1.48 | -1.96 ± 1.56 | 0.510 |
| Valeric acid | -4.77 ± 1.88 | -2.23 ± 1.98 | 0.496 |
| **Unbranched SCFA** | 40.83 ± 28.95 | -4.22 ± 30.57 | 0.433 |
| Iso-butyric acid | 8.79 ± 6.58 | -0.27 ± 6.95 | 0.488 |
| Iso-valeric acid | -4.70 ± 2.06 | -3.11 ± 2.17 | 0.698 |
| **Branched SCFA** | 4.09 ± 7.62 | -3.39 ± 8.05 | 0.620 |
| **Total SCFA** | 44.93 ± 35.69 | -7.61 ± 37.69 | 0.458 |
| **Indoles and phenols (µg/g)** | | | |
| Indole | 65.34 ± 29.55 | -47.22 ± 33.18 | 0.064 |
| Skatole | -20.46 ± 12.54 | -52.29 ± 14.08 | 0.213 |
| Phenol | 16.38 ± 10.28 | 10.40 ± 11.54 | 0.774 |
| *p*-cresol | -17.00 ± 50.65 | -260.61 ± 56.88 | 0.021 |
| **Total Indoles and phenols** | 44.27 ± 78.54 | -334.49 ± 86.33 | 0.019 |
| **Neutral sterol (µg/g)** | | | |
| Coprostanol | 29.45 ± 35.89 | 34.17 ± 35.89 | 0.946 |
| Coprostanone | 1.49 ± 1.09 | 1.31 ± 1.09 | 0.933 |
| Cholesterol | 38.00 ± 15.48 | 27.37 ± 15.48 | 0.725 |
| Cholestanol | 0.39 ± 0.88 | 1.84 ± 0.88 | 0.400 |
| Cholestanone | 0.10 ± 0.13 | 0.38 ± 0.13 | 0.280 |
| Cholestenone | 0.41 ± 0.12 | 0.12 ± 0.12 | 0.213 |
| **Total Neutral sterol** | 69.84 ± 39.65 | 65.20 ± 39.65 | 0.952 |
| **Bile acids (µg/g)** | | | |
| Cholic acid | -0.37 ± 0.24 | 0.37 ± 0.24 | 0.115 |
| Chenodeoxycholic acid | -0.17 ± 0.42 | 0.32 ± 0.42 | 0.550 |
| **Primary bile acid** | -0.54 ± 0.50 | 0.70 ± 0.50 | 0.205 |
| Lithocholic acid | 28.14 ± 26.74 | 33.85 ± 26.74 | 0.913 |
| Deoxycholic acid | 8.08 ± 2.86 | 2.05 ± 2.86 | 0.282 |
| Iso-lithocholic acid | 0.02 ± 0.33 | 0.01 ± 0.33 | 0.979 |
| Iso-deoxycholic acid | -0.01 ± 0.24 | 0.10 ± 0.24 | 0.800 |
| **Secondary bile aicd** | 36.23 ± 27.21 | 36.01 ± 27.21 | 0.997 |
| **Total bile acids** | 35.69 ± 27.20 | 36.70 ± 27.20 | 0.985 |
| ^1^ LSmean ± SE (all such values). LPQ, *Lactobacillus plantarum* Q180. ^2^ Linear mixed-effect model was used to analyze the effect of group*time for 12 weeks. | | | |
