# Supplementary material for: Effects of Lactobacillus plantarum Q180 on Postprandial Lipid Levels and Intestinal Environment: A Double-Blind, Randomized, Placebo-Controlled, Parallel Trial
Source: Nutrients. 2020 Jan 19;12(1):255. doi: 10.3390/nu12010255 (PMC7019774; doi:10.3390/nu12010255)
Supplement: Supplementary file 1 [file nutrients-12-00255-s001.zip › Supplement table 2.docx]

| **Supplementary Table S2.** The overall correlation between baseline levels of intestinal microbiota and changes in blood lipid markers for 12 weeks ^1^. | | | | | |
| --- | --- | --- | --- | --- | --- |
| Variables | | Placebo (n=31) | | LPQ 180 (n=31) | |
|  |  | r ^2^ | *p* | r ^2^ | *p* |
| *Streptococcus salivarius* | Δ TG | 0.223 | 0.443 | 0.251 | 0.174 |
|  | Δ TC | 0.306 | 0.094 | 0.127 | 0.495 |
|  | Δ LDL-C | 0.153 | 0.411 | -0.066 | 0.723 |
|  | Δ HDL-C | 0.171 | 0.359 | -0.094 | 0.617 |
|  | Δ VLDL-C | 0.131 | 0.482 | 0.251 | 0.174 |
|  | Δ Chylomicron TG | 0.204 | 0.235 | 0.212 | 0.252 |
|  | Δ ApoB | 0.220 | 0.557 | 0.128 | 0.492 |
|  | Δ ApoB-48 | 0.176 | 0.370 | 0.078 | 0.677 |
|  | Δ ApoB-100 | 0.188 | 0.338 | 0.223 | 0.228 |
|  | Δ Biogenic amines | 0.271 | 0.140 | 0.200 | 0.280 |
|  | Δ SCFAs | 0.163 | 0.382 | 0.508 | 0.004 |
|  | Δ Indole and phenols | 0.114 | 0.542 | 0.112 | 0.548 |
|  | Δ Neutral sterol | -0.049 | 0.792 | 0.073 | 0.697 |
|  | Δ primary bile acids | -0.228 | 0.218 | -0.520 | 0.003 |
|  | Δ secondary bile acids | -0.105 | 0.573 | -0.008 | 0.966 |
| *Klebsiella pneumoniae* | Δ TG | -0.070 | 0.812 | 0.104 | 0.579 |
|  | Δ TC | -0.013 | 0.946 | 0.170 | 0.360 |
|  | Δ LDL-C | -0.166 | 0.372 | 0.028 | 0.882 |
|  | Δ HDL-C | 0.045 | 0.810 | -0.171 | 0.359 |
|  | Δ VLDL-C | -0.057 | 0.760 | 0.104 | 0.579 |
|  | Δ Chylomicron TG | -0.035 | 0.861 | -0.150 | 0.422 |
|  | Δ ApoB | -0.046 | 0.808 | 0.092 | 0.622 |
|  | Δ ApoB-48 | -0.034 | 0.862 | 0.066 | 0.726 |
|  | Δ ApoB-100 | -0.073 | 0.712 | 0.102 | 0.585 |
|  | Δ Biogenic amines | -0.020 | 0.914 | 0.050 | 0.790 |
|  | Δ SCFAs | -0.040 | 0.831 | 0.057 | 0.762 |
|  | Δ Indole and phenols | 0.034 | 0.858 | -0.032 | 0.865 |
|  | Δ Neutral sterol | 0.153 | 0.410 | -0.015 | 0.936 |
|  | Δ primary bile acids | 0.076 | 0.684 | -0.020 | 0.917 |
|  | Δ secondary bile acids | -0.072 | 0.701 | 0.041 | 0.828 |
| *Romboutsia timonensis* | Δ TG | -0.045 | 0.880 | 0.040 | 0.832 |
|  | Δ TC | 0.009 | 0.960 | -0.054 | 0.774 |
|  | Δ LDL-C | -0.031 | 0.867 | -0.061 | 0.745 |
|  | Δ HDL-C | 0.050 | 0.789 | 0.061 | 0.747 |
|  | Δ VLDL-C | -0.046 | 0.805 | 0.040 | 0.832 |
|  | Δ Chylomicron TG | -0.070 | 0.722 | -0.015 | 0.945 |
|  | Δ ApoB | -0.079 | 0.674 | -0.059 | 0.755 |
|  | Δ ApoB-48 | 0.057 | 0.772 | 0.090 | 0.632 |
|  | Δ ApoB-100 | -0.115 | 0.560 | -0.004 | 0.984 |
|  | Δ Biogenic amines | -0.246 | 0.182 | 0.083 | 0.658 |
|  | Δ SCFAs | -0.190 | 0.307 | 0.091 | 0.626 |
|  | Δ Indole and phenols | -0.130 | 0.486 | -0.242 | 0.190 |
|  | Δ Neutral sterol | -0.264 | 0.152 | -0.149 | 0.423 |
|  | Δ primary bile acids | -0.068 | 0.715 | -0.132 | 0.481 |
|  | Δ secondary bile acids | -0.075 | 0.690 | 0.083 | 0.658 |
| *Ruminococcus callidus* | Δ TG | -0.034 | 0.909 | -0.057 | 0.762 |
|  | Δ TC | -0.354 | 0.051 | 0.216 | 0.243 |
|  | Δ LDL-C | -0.345 | 0.057 | 0.094 | 0.616 |
|  | Δ HDL-C | -0.140 | 0.452 | -0.039 | 0.836 |
|  | Δ VLDL-C | -0.019 | 0.919 | -0.057 | 0.762 |
|  | Δ Chylomicron TG | -0.057 | 0.774 | -0.053 | 0.777 |
|  | Δ ApoB | -0.377 | 0.037 | 0.218 | 0.240 |
|  | Δ ApoB-48 | 0.192 | 0.327 | 0.105 | 0.574 |
|  | Δ ApoB-100 | 0.154 | 0.435 | 0.028 | 0.881 |
|  | Δ Biogenic amines | -0.102 | 0.584 | -0.077 | 0.679 |
|  | Δ SCFAs | -0.014 | 0.940 | -0.171 | 0.356 |
|  | Δ Indole and phenols | 0.035 | 0.851 | 0.252 | 0.171 |
|  | Δ Neutral sterol | 0.157 | 0.399 | 0.007 | 0.972 |
|  | Δ primary bile acids | 0.133 | 0.475 | 0.441 | 0.013 |
|  | Δ secondary bile acids | 0.013 | 0.947 | -0.123 | 0.512 |
| *Flavonifractor plautii* | Δ TG | 0.356 | 0.212 | 0.010 | 0.956 |
|  | Δ TC | -0.103 | 0.583 | 0.188 | 0.312 |
|  | Δ LDL-C | -0.133 | 0.477 | 0.151 | 0.418 |
|  | Δ HDL-C | 0.174 | 0.351 | 0.414 | 0.021 |
|  | Δ VLDL-C | 0.156 | 0.402 | 0.010 | 0.956 |
|  | Δ Chylomicron TG | 0.083 | 0.673 | -0.122 | 0.512 |
|  | Δ ApoB | -0.163 | 0.380 | 0.203 | 0.274 |
|  | Δ ApoB-48 | 0.011 | 0.954 | -0.081 | 0.664 |
|  | Δ ApoB-100 | -0.157 | 0.426 | 0.078 | 0.677 |
|  | Δ Biogenic amines | 0.153 | 0.413 | 0.224 | 0.225 |
|  | Δ SCFAs | 0.082 | 0.661 | 0.089 | 0.634 |
|  | Δ Indole and phenols | -0.160 | 0.391 | -0.032 | 0.864 |
|  | Δ Neutral sterol | -0.051 | 0.787 | -0.051 | 0.787 |
|  | Δ primary bile acids | 0.014 | 0.942 | 0.052 | 0.783 |
|  | Δ secondary bile acids | -0.154 | 0.409 | -0.011 | 0.952 |
| *Kineothrix alysoides* | Δ TG | 0.078 | 0.791 | 0.027 | 0.886 |
|  | Δ TC | -0.012 | 0.950 | -0.345 | 0.058 |
|  | Δ LDL-C | -0.106 | 0.571 | -0.420 | 0.019 |
|  | Δ HDL-C | -0.104 | 0.579 | -0.187 | 0.314 |
|  | Δ VLDL-C | 0.085 | 0.650 | 0.027 | 0.886 |
|  | Δ Chylomicron TG | 0.097 | 0.622 | 0.327 | 0.073 |
|  | Δ ApoB | -0.081 | 0.665 | -0.299 | 0.103 |
|  | Δ ApoB-48 | 0.153 | 0.437 | 0.084 | 0.654 |
|  | Δ ApoB-100 | 0.281 | 0.147 | -0.024 | 0.897 |
|  | Δ Biogenic amines | 0.024 | 0.897 | 0.037 | 0.843 |
|  | Δ SCFAs | 0.026 | 0.892 | -0.143 | 0.445 |
|  | Δ Indole and phenols | 0.004 | 0.985 | 0.077 | 0.682 |
|  | Δ Neutral sterol | 0.258 | 0.161 | -0.172 | 0.356 |
|  | Δ primary bile acids | 0.169 | 0.363 | 0.146 | 0.435 |
|  | Δ secondary bile acids | 0.119 | 0.526 | -0.011 | 0.955 |
| *Intestinibacter bartlettii* | Δ TG | -0.087 | 0.768 | 0.099 | 0.596 |
|  | Δ TC | 0.120 | 0.519 | -0.028 | 0.882 |
|  | Δ LDL-C | 0.032 | 0.866 | -0.087 | 0.642 |
|  | Δ HDL-C | -0.003 | 0.988 | -0.193 | 0.298 |
|  | Δ VLDL-C | -0.076 | 0.684 | 0.099 | 0.596 |
|  | Δ Chylomicron TG | -0.071 | 0.720 | 0.155 | 0.406 |
|  | Δ ApoB | -0.017 | 0.927 | -0.060 | 0.749 |
|  | Δ ApoB-48 | -0.150 | 0.448 | 0.060 | 0.750 |
|  | Δ ApoB-100 | 0.118 | 0.550 | 0.037 | 0.844 |
|  | Δ Biogenic amines | -0.021 | 0.911 | 0.128 | 0.494 |
|  | Δ SCFAs | 0.120 | 0.519 | 0.140 | 0.452 |
|  | Δ Indole and phenols | 0.128 | 0.493 | 0.074 | 0.693 |
|  | Δ Neutral sterol | -0.039 | 0.835 | -0.003 | 0.989 |
|  | Δ primary bile acids | 0.114 | 0.543 | -0.080 | 0.670 |
|  | Δ secondary bile acids | -0.146 | 0.435 | -0.017 | 0.928 |
| *Rothia mucilaginosa* | Δ TG | 0.697 | 0.006 | 0.265 | 0.150 |
|  | Δ TC | 0.054 | 0.773 | 0.133 | 0.477 |
|  | Δ LDL-C | -0.107 | 0.568 | -0.029 | 0.878 |
|  | Δ HDL-C | 0.291 | 0.113 | 0.016 | 0.930 |
|  | Δ VLDL-C | 0.181 | 0.331 | 0.265 | 0.150 |
|  | Δ Chylomicron TG | 0.200 | 0.307 | 0.241 | 0.191 |
|  | Δ ApoB | 0.004 | 0.985 | 0.159 | 0.394 |
|  | Δ ApoB-48 | 0.192 | 0.328 | 0.060 | 0.750 |
|  | Δ ApoB-100 | -0.167 | 0.395 | 0.274 | 0.137 |
|  | Δ Biogenic amines | 0.123 | 0.511 | 0.201 | 0.279 |
|  | Δ SCFAs | 0.105 | 0.574 | 0.518 | 0.003 |
|  | Δ Indole and phenols | -0.040 | 0.830 | 0.086 | 0.646 |
|  | Δ Neutral sterol | -0.274 | 0.137 | 0.118 | 0.527 |
|  | Δ primary bile acids | -0.227 | 0.219 | -0.556 | 0.001 |
|  | Δ secondary bile acids | -0.081 | 0.665 | 0.021 | 0.912 |
| *Weissella confusa* | Δ TG | 0.071 | 0.810 | 0.139 | 0.455 |
|  | Δ TC | 0.149 | 0.425 | 0.199 | 0.284 |
|  | Δ LDL-C | 0.011 | 0.955 | 0.169 | 0.363 |
|  | Δ HDL-C | 0.148 | 0.426 | -0.239 | 0.196 |
|  | Δ VLDL-C | 0.063 | 0.735 | 0.139 | 0.455 |
|  | Δ Chylomicron TG | 0.085 | 0.669 | -0.147 | 0.429 |
|  | Δ ApoB | 0.064 | 0.732 | 0.141 | 0.450 |
|  | Δ ApoB-48 | 0.080 | 0.687 | 0.062 | 0.740 |
|  | Δ ApoB-100 | 0.079 | 0.688 | 0.204 | 0.270 |
|  | Δ Biogenic amines | -0.206 | 0.267 | -0.206 | 0.266 |
|  | Δ SCFAs | -0.290 | 0.114 | 0.099 | 0.596 |
|  | Δ Indole and phenols | -0.119 | 0.524 | -0.024 | 0.900 |
|  | Δ Neutral sterol | -0.231 | 0.211 | 0.562 | 0.001 |
|  | Δ primary bile acids | 0.114 | 0.541 | -0.088 | 0.639 |
|  | Δ secondary bile acids | 0.009 | 0.963 | 0.162 | 0.383 |
| *Ruminococcus bromii* | Δ TG | 0.118 | 0.689 | -0.325 | 0.075 |
|  | Δ TC | 0.119 | 0.523 | -0.327 | 0.073 |
|  | Δ LDL-C | -0.077 | 0.681 | -0.184 | 0.321 |
|  | Δ HDL-C | 0.272 | 0.140 | 0.070 | 0.710 |
|  | Δ VLDL-C | 0.140 | 0.452 | -0.325 | 0.075 |
|  | Δ Chylomicron TG | 0.215 | 0.271 | -0.093 | 0.619 |
|  | Δ ApoB | 0.032 | 0.864 | -0.331 | 0.069 |
|  | Δ ApoB-48 | 0.179 | 0.362 | -0.170 | 0.361 |
|  | Δ ApoB-100 | -0.017 | 0.931 | -0.209 | 0.260 |
|  | Δ Biogenic amines | 0.173 | 0.352 | 0.235 | 0.204 |
|  | Δ SCFAs | 0.089 | 0.635 | 0.052 | 0.781 |
|  | Δ Indole and phenols | 0.017 | 0.929 | -0.447 | 0.012 |
|  | Δ Neutral sterol | 0.275 | 0.134 | -0.256 | 0.165 |
|  | Δ primary bile acids | 0.231 | 0.210 | -0.123 | 0.509 |
|  | Δ secondary bile acids | -0.271 | 0.140 | -0.023 | 0.904 |
| *Flintibacter butyricus* | Δ TG | 0.034 | 0.908 | -0.304 | 0.096 |
|  | Δ TC | 0.134 | 0.473 | -0.083 | 0.657 |
|  | Δ LDL-C | 0.170 | 0.361 | 0.038 | 0.839 |
|  | Δ HDL-C | 0.141 | 0.449 | 0.078 | 0.678 |
|  | Δ VLDL-C | 0.125 | 0.503 | -0.304 | 0.096 |
|  | Δ Chylomicron TG | 0.014 | 0.944 | -0.138 | 0.460 |
|  | Δ ApoB | 0.201 | 0.279 | -0.051 | 0.788 |
|  | Δ ApoB-48 | 0.171 | 0.385 | 0.062 | 0.742 |
|  | Δ ApoB-100 | 0.072 | 0.718 | -0.151 | 0.419 |
|  | Δ Biogenic amines | 0.080 | 0.671 | -0.016 | 0.930 |
|  | Δ SCFAs | -0.073 | 0.696 | -0.121 | 0.516 |
|  | Δ Indole and phenols | 0.281 | 0.126 | -0.152 | 0.416 |
|  | Δ Neutral sterol | 0.471 | 0.007 | -0.109 | 0.560 |
|  | Δ primary bile acids | 0.168 | 0.367 | 0.017 | 0.926 |
|  | Δ secondary bile acids | -0.257 | 0.162 | 0.101 | 0.590 |
| *Barnesiella intestinihominis* | Δ TG | 0.040 | 0.892 | -0.407 | 0.023 |
|  | Δ TC | -0.349 | 0.054 | -0.138 | 0.458 |
|  | Δ LDL-C | -0.357 | 0.049 | -0.038 | 0.841 |
|  | Δ HDL-C | -0.332 | 0.068 | -0.076 | 0.686 |
|  | Δ VLDL-C | 0.056 | 0.765 | -0.407 | 0.023 |
|  | Δ Chylomicron TG | 0.177 | 0.367 | -0.141 | 0.450 |
|  | Δ ApoB | -0.361 | 0.046 | -0.153 | 0.410 |
|  | Δ ApoB-48 | 0.328 | 0.089 | 0.036 | 0.847 |
|  | Δ ApoB-100 | 0.499 | 0.007 | -0.002 | 0.990 |
|  | Δ Biogenic amines | -0.056 | 0.766 | -0.317 | 0.082 |
|  | Δ SCFAs | -0.076 | 0.685 | -0.567 | 0.001 |
|  | Δ Indole and phenols | -0.029 | 0.878 | 0.014 | 0.942 |
|  | Δ Neutral sterol | 0.061 | 0.743 | -0.152 | 0.416 |
|  | Δ primary bile acids | 0.120 | 0.520 | 0.575 | 0.001 |
|  | Δ secondary bile acids | -0.068 | 0.717 | 0.316 | 0.083 |
| ^1^ LPQ 180, Lactobacillus plantarum Q180. ^2^ r : Pearson's correlation coefficients | | | | | |
